# Adolescent Binge Drinking in the West of Ireland: Associated Risk and Protective Factors

**DOI:** 10.1093/eurpub/ckac129.519

**Published:** 2022-10-25

**Authors:** C Kelly, Á McNamara

**Affiliations:** 1 Health Protection Surveillance Centre, Dublin, Ireland; 2 Department of Public Health, HSE West, Galway, Ireland

## Abstract

This study aimed to examine potential risk and protective factors for binge drinking among a cohort of 15-16-year-old adolescents in the West of Ireland. This study was a cross-sectional secondary analysis of 4,473 15-16-year-olds who participated in the 2020 Planet Youth survey. Binge drinking was defined as ever consumption of five or more drinks in a two-hour period or less. Data were analysed using SPSS version 27. Multivariable logistic regression was used to examine independent associations between potential risk and protective factors and binge drinking. A p-value of < 0.05 was deemed statistically significant. The prevalence of binge drinking among participants was 34.1%. Female gender (aOR 0.55, 95% CI 0.46-0.67, p < 0.001) and non-White ethnicity (aOR 0.49, 95% CI 0.31-0.77, p = 0.002) were associated with reduced odds of ever binge drinking. Self-rated ‘bad/very bad’ mental health (aOR 1.61, 95% CI 1.26-2.06, p < 0.001), current cigarette use (aOR 4.06, 95% CI 3.01-5.47, p < 0.001) and current cannabis use (aOR 2.79, 95% CI 1.80-4.31, p < 0.001) were associated with increased odds of ever binge drinking. Parental supervision (aOR 0.80, 95% CI 0.73-0.88, p < 0.001) and negative parental reaction to adolescent drunkenness (aOR 0.51, 95% CI 0.42-0.61, p < 0.001) reduced the odds of ever binge drinking among participants. Getting alcohol from parents was associated with increased odds of ever binge drinking (aOR 1.79, 95% CI 1.42-2.25, p < 0.001). Adolescents with friends who drink alcohol had almost 5 times higher odds of ever binge drinking (aOR 4.59, 95% CI 2.65-7.94, p < 0.001). Participating in team sports was also associated with increased odds of ever binge drinking (aOR 1.30, 95% CI 1.07-1.57, p = 0.008 for 1-4 times/week, aOR 1.52, 95% CI 1.07-2.16, p = 0.020 for ≥5 times/week). This study highlights key influences of adolescents’ social environment on their binge drinking, and a need for renewed public health efforts to protect adolescents from alcohol-related harm.

**Key messages:**

• This study identified a high prevalence of ever binge drinking among adolescents in the West of Ireland - this is highly concerning as adolescents are vulnerable to alcohol-related harm.

• This study identified factors in the social environment of adolescents associated with binge drinking. This can inform public health action to protect adolescents from alcohol-related harm.

